# Prevalence and associated risk factors of Bovine mastitis in dairy cows in and around Assosa town, Benishangul‐Gumuz Regional State, Western Ethiopia

**DOI:** 10.1002/vms3.454

**Published:** 2021-03-02

**Authors:** Melak Tezera, Endris Aman Ali

**Affiliations:** ^1^ Department of Veterinary Science College of Agriculture and Environmental Science Assosa University Assosa Ethiopia; ^2^ Dabat District Livestock Resource Office Gondar Ethiopia; ^3^ Department of Veterinary Science Bahir Dar University Bahir Dar Ethiopia

**Keywords:** Assosa, dairy cows, ethiopia, mastitis, prevalence, risk factor

## Abstract

**Background:**

Mastitis, a complex disease of multifactorial aetiology, is one of the most costly diseases in the dairy industry worldwide. It can be categorized as clinical and subclinical type relying on the clinical sign. The objectives of the study were to determine the prevalence of mastitis and to identify its intrinsic and extrinsic risk factors in dairy cows in and around Assosa town, Western Ethiopia.

**Methods:**

A cross‐sectional study design was followed to address the objectives of the study. A total of 367 lactating cows were selected using simple random and systematic sampling techniques. Thorough clinical examination and California Mastitis Test (CMT) were deployed for detection of both clinical and subclinical mastitis, respectively.

**Results:**

Based on CMT result and clinical examination the cow level prevalence of mastitis was 40.3% (*n* = 148), of which 11.99% (*n* = 44) and 28.34% (*n* = 104) were clinical and subclinical mastitis respectively. The corresponding quarter‐level prevalence was determined to be 26.9% (*n* = 394), comprising 11.99% (*n* = 176) clinical and 14.85% (*n* = 218) subclinical mastitis. The Chi‐square analysis of intrinsic risk factors revealed statistically significant differences (*p* <.05) in the prevalence of mastitis among breed, stage of lactation and body condition score. Likewise, production system, previous mastitis exposure, hygiene practice and type of floor were extrinsic risk factors significantly associated with the occurrence of mastitis.

**Conclusions:**

In general, this study revealed a high prevalence of bovine mastitis in the study area. Thus, the current study shows the need for applying feasible mastitis intervention strategy with special emphasis on sub‐clinical mastitis and associated risk factors.

## Abstract 2

A cross‐sectional study design was followed to determine the prevalence and Associated risk factors of bovine mastitis in dairy cows in and around Assosa town. Based on clinical examination and CMT result the prevalence of mastitis was determined to be 40.3%.

## 1 INTRODUCTION

Mastitis is an inflammation of the parenchyma of mammary gland irrespective of the aetiology but the majority of the causes are infectious agents (Quinn et al., 2002). The disease is characterized by physical, chemical and bacteriological changes in milk and pathological changes in the glandular tissue that affects both the normal flow and quality of milk (Radostitis et al., 2007).

Despite mastitis is considered as a complex and multi factorial disease, bacterial pathogens share the greatest contributions. Based on distinct characteristics of distribution and interaction with teats and ducts, causative pathogenic microorganisms are classified as contagious and environmental pathogens. The major causes of contagious mastitis, which could be cow‐associated pathogens, includes *Streptococcus agalactiae* and *Staphylococcus aureus* while, *Streptococcus dysgalactiae*, *Streptococcus uberis* and *Escherichia coli* are the main causes of environmental mastitis (Smith, 2002).

According to Quinn et al. (2002) and Andrews et al. (2003) mastitis can also be classified as clinical and subclinical type relying on the manifestation of the disease. Clinical mastitis is when milk appears abnormal with chemical and physical changes. The mammary gland also may exhibit change in its morphology. The subclinical form occurs when both milk and udder appears normal without noticeable manifestations of inflammation. Subclinical mastitis is more prevalent than clinical mastitis and causes the greatest overall losses in most dairy herds worldwide (Eriskine, 2001).

Most estimates have shown that mastitic cow result in a 30% reduction in productivity per affected quarter and a 15% reduction in productivity per cow/lactation, making the disease one among the most costly diseases of dairy industry worldwide (Schalm et al., 1971). In addition, the bacterial contamination of milk from affected cows may render it unsuitable for human consumption due to zoonosis, food poisoning and antibiotic residue in the milk following mastitis (Radostitis et al., 2007).

Ethiopia has the largest livestock population in Africa with an estimated cattle population of 59.5 million, which contributes 40% of the annual agricultural output and 15% total gross domestic product. The annual milk and meat produced from cattle is 1.5 and 0.331 million tonnes, respectively. Cows represent the biggest portion of cattle population of the country, accounting 42% of the total cattle population (CSA, 2016/17). Despite the country has huge cattle population, its milk requirement and demand is not satisfied yet due to a multitude of factors. Mastitis is among the various factors contributing to reduced milk production (Biffa et al., 2005).

In Ethiopia, a number of researchers have studied the occurrence of mastitis in dairy cows. According to the most recent published studies, the cow‐level mastitis prevalence estimate falls within the range of 23.2% and 81.1% for the country (Abera et al., 2013; Endale et al., 2016; Sarba & Tola, 2017; Zeryehun et al., 2013). However, most of these research are carried out in and around Addis Ababa, which might not represent the occurrence of mastitis under different management and environmental situations in other regions of the country. Moreover there is few published data on the current status of mastitis in and around Assosa (Asmelash et al., 2017). Therefore, this study was aimed to determine the prevalence of bovine mastitis and to identify the potential intrinsic and extrinsic risk factors associated with the prevalence of bovine mastitis in dairy cows in and around Assosa town.

## 2 MATERIALS AND METHODS

### 2.1 Description of the study Area

This study was conducted between March 2019 and June 2019 in and around Assosa town. Assosa is the capital of Benishangul‐Gumuz Regional State and composed of 74 administrative peasant associations. It is located at 8°30’and 40°27′ N latitude and 34°21′ and 39°1′ E longitude, 687 km Northwest of Addis Ababa (CSA, 2015). The altitude of Assosa ranges from 580 to over 1,560 m above sea level. The area is characterized by low land plane agro‐ecology which has ‘kola’ micro climate (semi‐desert weather condition) with land coverage of 2,317 km^2^ areas. According to national meteorological service agency the average annual rainfall of the town is 850–1316 mm with uni‐modal type of rainfall that occurs between April and October. Its mean annual temperature ranges between 16.75°C and 36°C. The study area owns 35.6% of the livestock population of the region constituting 61, 234 cattle, 19,183 goats, 19,729 sheep, 25,137 donkeys, 439,969 poultry and 73,495 beehives (CSA, 2015). The study area comprises two districts and 10 ‘kebeles’ (the smallest administrative unit) and 7 kebeles were included in the study.

### 2.2 Study animals

The study populations were all lactating cows in and around Assosa town. Lactating dairy cattle (mostly indigenous zebu cattle from surrounding areas of Assosa) and Holstein‐Zebu cross from the small holder dairy farms in Assosa town constitutes study animals. Individual animal was selected randomly and examined for mastitis using clinical examinations and California Mastitis Test (CMT).

### 2.3 Study design

A cross‐sectional type of study was followed to determine the prevalence of mastitis, and to identify its intrinsic and extrinsic risk factors in dairy cows in and around Assosa town, Western Ethiopia. The sample size was calculated based on the formula of Thrusfield (2005) at 95% confidence interval, 5% absolute precision and with expected prevalence of 39.32% (Asmelash et al., 2017). A total of 367 lactating cows; 213 Zebu and 154 Holstein‐Zebu cross were sampled. In this study, two types of sampling techniques (for intensive and extensive production systems) were used. In the intensive production system, 25 farms, with farm size between 12 and 25, were included in the study. All farms did not have registration practice, the farmers uses their own identification system (naming each cow). Based on the farmer's information we gave numbers for each cow. Then, 123 dairy cows were selected using simple random sampling by lottery method. In the extensive production system, we used a systematic sampling method. First, we selected seven ‘kebeles’ from a total of 10 kebeles using a simple random method. Then from these seven kebeles villages which owned dairy cows were sampled at equal intervals. Proportion was made to include samples from each kebeles. Accordingly, 35 dairy cows were sampled from six kebeles and the remaining 34 from 1 kebele. In total, 244 dairy cows were selected. Cows were examined clinically and tested for mastitis screening with CMT.

### 2.4 Data collection

A structured questioner with close ended questions was used to collect data about various cow and quarter level factors thought to impact the occurrence of mastitis in the dairy cows. The intrinsic factors like breed, age, body condition score (BCS), parity, stage of lactation and extrinsic factors such as production system, floor type, hygienic practice, tick infestation and previous clinical mastitis history were assessed to determine their association with the occurrence of mastitis.

The age of animals was determined by counting the number of rings in the horn and dentition characteristics and categorized as young adults (3–6 years), adults (6 to ≤10 years) and old (>10 year). Stage of lactation was categorized as early (1–4 months), middle (>4–8months), and late (>8 months to the beginning of dry period). Parity was also categorized as few (with ≤3calves), moderate (4–7 calves), and many (>7 calves) and BCS was categorized as poor (if the dairy cows are emaciated with visible ribs from distance) and good (if the dairy cows are well nourished with at least no visible ribs from distance). The floor was grouped into muddy (floor which was not well managed) and concrete (floor which is well managed). Milking hygiene practice was categorized into good (if there is a practice of washing and drying udder with separate towels, milking healthy and young cows first) and poor (If washing and drying of udder with a separate towel and milking with order is not practiced) and production system was also categorized into intensive (if the farms have feed reserves sufficient for a year,operated by a hired labour and thus, cows are managed with intensive feeding and watering) and extensive ( if the farms have not feed reserves for a year, operated mainly by family labour and thus, cows are allowed to graze in the field extensively; Biffa et al., 2005).

### 2.5 Milk sample collection

Procedure for collection of milk was according to Quinn et al. (2002). Milk sampling and screening were performed for each quarter. The time chosen for milk sampling was before milking. Information on the cow age, breed, parity, lactation stage, BCS, production system, floor type, milking hygienic practice, tick infestation and previous mastitis history were also collected at the time of sampling using data recording sheet.

### 2.6 Diagnosis of mastitis

In this study, the lactating cow's udder and teats were clinically examined to know the abnormalities before milk sample collection. Clinical examination was undertaken according to Radostitis et al. (2007). Accordingly, each cows udder was carefully inspected to check the symmetry of quarters followed by thorough palpation to detect possible fibrosis, inflammatory swellings, visible injury, tick infestation, atrophy of the tissue and swelling of supra mammary lymph nodes. Furthermore, viscosity and appearance of milk secretion from each mammary quarter were examined for the presence of clots, flakes, blood and watery secretions (Biffa et al., 2005). The presence of clots, flakes, blood and other consistency changes were part of the indicators for clinical mastitis along with udder and teat morphological changes.

The California mastitis test (CMT) was used to screen cows with sub‐clinical mastitis. The collected milk samples were screened by the CMT according to Michael. (2011). From each quarter of the udder, a squirt of milk was placed in each of the four shallow cups on the CMT paddle, and an equal amount of CMT reagent manufactured in Poland, which is commercially available Ethiopia, was added to each cup. A gentle circular motion was applied to the mixtures in a horizontal plane for 15 s. The result of CMT was based on the nature of coagulation and viscosity of the mixture which show the presence and severity of the infection, respectively (Fufa et al., 2013). The result was inferred based on the thickness of gel formed by CMT reagent and milk mixture and scored as 0 (none), trace (very mild), 1 (mild), 2 (moderate) and 3 (heavy, almost solid). Finally, quarters with CMT score of 1 or above was considered as positive for sub clinical mastitis; otherwise negative (Michael, 2011).

### 2.7 Data management and analysis

Collected data were first entered into Microsoft Excel spreadsheet and analysed using Statistical Package for Social Sciences (SPSS) statistical software version 16.0. Descriptive measures like proportions and percentages were used to summarize and present the data collected. The prevalence of mastitis was calculated as the number of lactating cows tested positive by CMT test or cows showing symptoms of clinical mastitis, divided by the total number of tested or clinically examined animals.

The existence of association between independent variables with the occurrence of dependent variables (mastitis) was verified using Pearson Chi‐square (*χ* ^2^) statistical test. The independent variables assessed were production system, floor type, breed, age, BCS, parity, stage of lactation, hygienic practice, tick infestation and previous clinical mastitis history. For this study, the level of significance was set at 0.05 with 95% confidence interval. The results of this study were considered statistically significant when *p* <.05.

## 3 RESULTS

### 3.1 Overall prevalence of mastitis

From a total of 367 cows the overall prevalence of mastitis at cow level as determined by CMT and clinical examination was 40.3% (*n* = 148). Out of this, the prevalence of clinical and subclinical mastitis is determined to be 11.99% (*n* = 44) and 28.34% (*n* = 104), respectively. Of the 1,468 quarters examined 3.9% (*n* = 57) teats were found blind. From the functional 1,411 teats examined 11.99% (*n* = 176) quarters showed clinical mastitis. From those teats screened by CMT 14.85% (*n* = 218) quarters showed evidence of subclinical mastitis (Table 1).

**TABLE 1:** Prevalence of mastitis at cow and quarter level in dairy cows of Assosa town

Type of mastitis	No of cows examined	No of cows with mastitis (%)	Number of Quarter examined	Number of quarter with mastitis (%)
Clinical	367	44/367 (11.99%)	1,468	176/1468 (11.99%)
Subclinical	367	104/367 (28.34%)	1,468	218/1468 (14.85%)
Blind teat	367	—	1,468	57/1468 (3.9%)
Total	367	148	1,468	451/1468 (30.72%)

### 3.2 Risk factors

This study revealed significantly higher prevalence of mastitis 50% (*n* = 77) in cross breeds as compared to local breeds 33.3% (*n* = 71; *p* <.05; Table 2). In comparing prevalence among production systems, a statistically significant (*p* <.05) peak prevalence was observed in cows reared under intensive production system 54.4% (*n* = 67) than extensive production system 33.1% (*n* = 81; Table 3). In this study, cows affected with clinical mastitis previously, were found to be more prone to mastitis than non‐exposed ones with prevalence of 66.3% (*n* = 61) and 31.6% (*n* = 87), respectively, and the result is statistically significant (*p* <.05; Table 3).

**TABLE 2:** Prevalence of mastitis in milking cows based on intrinsic risk factors

Intrinsic factors	Category	No of cows examined	No of cows with mastitis (%)		*χ* ^2^	*p*‐value
Age	Young‐adult (3–6 year)	114	44/114 (38.5%)	4.043		.189
	Adult (>6–10 year)	123	46/123 (37.3%)			
	Old (>10 year)	130	58/130 (44.6%)			
Breed	Local	213	71/213 (33.3%)	20.062		.000
	Cross	154	77/154 (50% )			
Parity	Few	125	42/125 (33.6%)	4.043		.132
	Moderate	119	47/119 (39.4%)			
	Many	123	59/123 (47.9%)			
Lactation stage	Early (1–4 month)	128	72/128 (56.2%)	24.36		.001
	Middle (>4–8 month)	131	27/131 (20.6%)			
	Late(>8 month)	108	49/108 (45.3%)			
Body condition score	Good	239	81/239 (33.8%)	10.141		.001
	Poor	128	67/128 (52.3%)			

**TABLE 3:** Prevalence of mastitis in milking cows based on extrinsic risk factors

Extrinsic risk factors	Category	No of cows examined	No of cows with mastitis (%)	*χ* ^2^	*p*‐value
Floor type	Concrete	242	81/242 (33.4%)	9.077	.003
	Muddy	125	67/125 (53.6%)		
Production system	Extensive	244	81/244 (33.1%)	10.357	.001
	Intensive	123	67 /123 (54.4%)		
Hygienic practice	Good	236	83/236 (33.1%)	6.946	.008
	Poor	131	65/131 (49.6%)		
Clinical mastitis history	Yes	92	61/92 (66.3%)	24.636	.00
	No	275	87/275 (31.6%)		
Tick infestation	Yes	127	59/127 (46.4%)	1.640	.200
	No	240	89/240 (37%)		

Lactation stage was considered as risk factor for the occurrence of mastitis in this report. Cows during early stage of lactation had a higher mastitis prevalence 56.2% (*n* = 72) than middle 20.6% (*n* = 27) and late 45.3% (*n* = 49) stage of lactation and the difference was statistically significant (*p* <.05; Table 2). Generally in this study breed, production systems, previous clinical mastitis history, lactation stage, BCS, hygiene practice and floor type were found significantly associated with the occurrence of mastitis. However, in our study there was no statistically significant (*p* >.05) difference in prevalence of mastitis between animals age, parity and status of tick infestation (Table 2 and 3).

## 4 DISCUSSION

The study was conducted on bovine mastitis in Assosa and its surroundings to determine the prevalence, and major intrinsic and extrinsic factors associated with the disease. The result revealed an overall prevalence of 40.3% in the study area, which is in agreement with 40% prevalence reports of Kerros and Tareke (2003), and 40.4% of Dego and Tareke (2003) from southern Ethiopia. The current finding of this study is slightly higher than that of Asmelash et al. (2017), who reported an overall prevalence of 39.32% in Assosa town.

However, our study result is comparably very low when compared with the prevalence report of 63.11% by Kassa et al. (2014) in Hawassa and Wando Genet, 46.7% by Abera et al. (2013) in Adama, 63.02%, by Biniam et al. (2019) in Haramaya district, 71.05% by Mekibib et al. (2010) in Holeta town of central Ethiopia and 74.7% by Zeryehun et al. (2013) in and around Addis Ababa. Our study finding is higher than the prevalence report of Bitew et al. (2010), Mulugeta and Wassie, (2013) and Endale et al. (2016), who had reported the prevalence of mastitis as 28.8%, 29.5% and 32.92% in Bahir Dar, Wolayita Sodo and Southern Ethiopia, respectively. The reason for the disagreement between the current and the previous studies could be due to the difference in the management systems, breeds of cattle and agro climatic areas, which could contribute to the variability of mastitis prevalence among reports.

The prevalence of clinical mastitis recorded in the present study is 14.9% and that of subclinical mastitis 28.33%, which is by far higher than the occurrence of clinical cases. This finding is in line with Bitew et al. (2010), who reported clinical prevalence of 3% and also subclinical cases of 25.2% at Bahir Dar and its environs. As it can be clearly seen in most published articles report (including the present finding) clinical mastitis is far lower than subclinical mastitis (Almaw et al., 2008; Biffa et al., 2005; Haftu et al., 2012; Lakew et al., 2009; Mulugeta & Wassie, 2013; Sori et al., 2005). This could be attributed to the little attention given to subclinical mastitis, as the infected animals show no obvious symptoms and secrets apparently normal milk and farmers especially, small holders are not well informed about invisible loss from subclinical mastitis. In Ethiopia, little attention is given to the subclinical forms of mastitis and efforts have been concentrated on the treatment of clinical cases (Almaw et al., 2008; Mulugeta & Wassie, 2013).

The quarter level mastitis prevalence (26.9%) recorded in the current study is higher as compared to previous reports (Almaw et al., 2008; Bitew et al. 2010; Haftu et al.,^2012^; Lakew et al., 2009). The finding of 3.9% blind teat in this work is in agreement with the previous report of Biffa et al. (2005) and higher when compared with the reports of Haftu et al. (2012) and Bitew et al. (2010). The high proportion of blind quarters observed in this study might indicate the serious impact imposed by mastitis in the study area and the absence of culling that should have served to remove a source of mammary pathogens for the cows. Besides, unable to screen subclinical mastitis and late or complete absence of treating clinical cases could possibly leads to blindness of mammary quarter. It can be hypothesized that blind mammary quarters contribute to high subclinical mastitis and loss of milk production with a subsequent impact on food security.

The current study showed the significant association between prevalence of mastitis with breeds. Accordingly, cross breed cows are more likely affected by mastitis than that of local breed cows. This finding is in agreement with Sori et al. (2005), Moges et al. (2012) and Kassa et al. (2014), who have explained that genetic predisposition factors to mastitis such as teat shape, sphincter tone, anatomy of the teat canal and susceptibility to weakening of the suspensory ligament (pendulous udder). However, the discrepancy of mastitis prevalence among breeds might reflect the differences in management practice rather than a true genetic difference (Radostitis et al., 2007).

The occurrence of bovine mastitis and lactation stage was significantly (*p* <.05) associated. That is, higher infection in cows in early lactation stage followed by late and medium lactation stages, that concurs with previous reports (Biffa et al., 2005; Mulugeta & Wassie, 2013). The early lactation stage infection might be due to the carryover of infection from dry period. In dairy cows most new infections occur during the early part of the dry period and in the first two months of lactation (Radostits et al., 2007).

The current finding showed significant higher prevalence of bovine mastitis in intensive production systems (54.4%) when compared with extensive production systems (33.3%). This could be attributed to the variation in hygienic standards of dairy environment and milking conditions. In most dairy farms cows kept in intensive system maintained in a dirty and wet area which could favours the proliferation and transmission of mastitis causing organisms. Under Ethiopian condition dairy farmers have no experience to exercise management practices such as teat dipping, milking with gloves and machine milking.

The prevalence of mastitis in cows kept under poor hygiene and cows with good hygienic status was found to be 49.6% and 35.1%, respectively. Prevalence of mastitis was significantly (*p* <.05) associated with milking hygienic practice. Dairy cows with poor milking hygiene standard are severely affected than those with good milking hygiene practices (Abera et al. (2013; Lakew et al., 2009; Sori et al., 2005). This might be due to absence of udder washing, milking of cows with common milkers’ and using of common udder cloths, which could serve as a vehicle to spread especially for contagious mastitis.

## 5 CONCLUSIONS

The present study has shown that mastitis, particularly sub‐clinical type, is a widely prevalent disease of dairy cows in and around Assosa town both at cow and quarter level. Majority of the risk factors noted are the main reasons for the observed high prevalence of mastitis in the study area. Accordingly, cross‐breed cows at early stage of lactation which had poor body condition score were at higher risk of contracting mastitis. Besides, the presence of previous clinical mastitis history and keeping dairy cows under intensive production system in muddy floor with poor hygienic practice were also noted to favour the occurrence of mastitis significantly. Therefore, the current study indicates/implicates the need for applying feasible mastitis intervention strategy with special emphasis on sub‐clinical mastitis. Since our study was only prevalence based further detailed epidemiological, microbiological and economic analysis studies are suggested at countrywide level to shape the existing control and prevention strategies.

## AUTHOR CONTRIBUTIONS

**Endris ali:** Conceptualization; Data curation; Formal analysis; Methodology; Resources; Software; Writing‐review & editing. **Melak Tezera:** Conceptualization; Data curation; Investigation; Methodology; Resources; Visualization; Writing‐original draft.

### PEER REVIEW

The peer review history for this article is available at https://publons.com/publon/10.1002/vms3.454.
